# Direct prediction of genetic aberrations from pathology images in gastric cancer with swarm learning

**DOI:** 10.1007/s10120-022-01347-0

**Published:** 2022-10-20

**Authors:** Oliver Lester Saldanha, Hannah Sophie Muti, Heike I. Grabsch, Rupert Langer, Bastian Dislich, Meike Kohlruss, Gisela Keller, Marko van Treeck, Katherine Jane Hewitt, Fiona R. Kolbinger, Gregory Patrick Veldhuizen, Peter Boor, Sebastian Foersch, Daniel Truhn, Jakob Nikolas Kather

**Affiliations:** 1 Department of Medicine III, University Hospital RWTH Aachen, Aachen, Germany; 2 Else Kroener Fresenius Center for Digital Health, Medical Faculty Carl Gustav Carus, Technical University Dresden, Fetscherstrasse 74, 01307 Dresden, Germany; 3 Pathology and GROW School for Oncology and Developmental Biology, Maastricht University Medical Center+, Maastricht, The Netherlands; 4 Pathology and Data Analytics, Leeds Institute of Medical Research at St James’s, University of Leeds, Leeds, UK; 5 Institute of Pathology, Inselspital, University of Bern, Bern, Switzerland; 6 Institute of Pathology and Molecular Pathology, Kepler University Hospital, Johannes Kepler University Linz, Linz, Austria; 7 Institute of Pathology, TUM School of Medicine, Technical University of Munich, Munich, Germany; 8 Department of Visceral, Thoracic and Vascular Surgery, University Hospital and Faculty of Medicine Carl Gustav Carus, Technische Universität Dresden, Dresden, Germany; 9 Institute of Pathology, University Hospital RWTH Aachen, 52074 Aachen, Germany; 10 Department of Nephrology and Immunology, University Hospital RWTH Aachen, 52074 Aachen, Germany; 11 Institute of Pathology, University Medical Center Mainz, Mainz, Germany; 12 Department of Diagnostic and Interventional Radiology, University Hospital RWTH Aachen, Aachen, Germany; 13 Medical Oncology, National Center for Tumor Diseases (NCT), University Hospital Heidelberg, Heidelberg, Germany; 14 Department of Medicine 1, University Hospital and Faculty of Medicine Carl Gustav Carus, Technische Universität Dresden, Dresden, Germany

**Keywords:** Gastric cancer, Pathology, Biomarker, Artificial intelligence, Blockchain, Swarm learning

## Abstract

**Background:**

Computational pathology uses deep learning (DL) to extract biomarkers from routine pathology slides. Large multicentric datasets improve performance, but such datasets are scarce for gastric cancer. This limitation could be overcome by Swarm Learning (SL).

**Methods:**

Here, we report the results of a multicentric retrospective study of SL for prediction of molecular biomarkers in gastric cancer. We collected tissue samples with known microsatellite instability (MSI) and Epstein–Barr Virus (EBV) status from four patient cohorts from Switzerland, Germany, the UK and the USA, storing each dataset on a physically separate computer.

**Results:**

On an external validation cohort, the SL-based classifier reached an area under the receiver operating curve (AUROC) of 0.8092 (± 0.0132) for MSI prediction and 0.8372 (± 0.0179) for EBV prediction. The centralized model, which was trained on all datasets on a single computer, reached a similar performance.

**Conclusions:**

Our findings demonstrate the feasibility of SL-based molecular biomarkers in gastric cancer. In the future, SL could be used for collaborative training and, thus, improve the performance of these biomarkers. This may ultimately result in clinical-grade performance and generalizability.

**Supplementary Information:**

The online version contains supplementary material available at 10.1007/s10120-022-01347-0.

## Introduction

Computational pathology refers to the use of deep learning (DL) methods in histopathology [1, 2]. DL can predict molecular biomarkers directly from routine tissue slides, which could be a helpful tool in precision oncology of solid tumors [3, 4]. Several molecular biomarkers are used to guide treatment in advanced and metastatic gastric cancer. In addition to HER2 and PD-L1 expression, which are clinically approved biomarkers for targeted treatment or immunotherapy in gastric cancer, microsatellite instability (MSI) and Epstein–Barr Virus (EBV) positivity have been linked to immunotherapy response [5]. Computational pathology can predict these biomarkers directly from pathology slides stained with hematoxylin and eosin (H&E), albeit with a lower performance than the diagnostic gold standard methods [6–10]. If MSI and EBV could be predicted from pathology slides with a sufficiently high sensitivity, this could improve clinical care and reduce costs [11]. While MSI status can be predicted from pathology slides with clinical-grade performance in colorectal cancer [7, 12], this seems more difficult in gastric cancer [13, 14]. In general, computer-based prediction of molecular biomarkers for treatment recommendation appears to be more complex in gastric cancer than in other tumor types. A possible reason for this lower performance is the histopathological heterogeneity. Unlike in colorectal cancer and other tumors of the digestive tract, gastric cancer can display very different histopathological growth patterns within the same specimen, which require skill and experience to diagnose. Consequently, multicentric studies for the detection of microsatellite instability (MSI) in gastric cancer have resulted in a lower performance than similar studies in colorectal cancer [12, 13]. In addition, gastric cancer has a highly heterogeneous geographic distribution, with high incidence regions clustered in South America, Eastern Europe, and central and East Asia. Investigators are not necessarily located in these regions, which necessitates an increased data sharing between institutions working on gastric cancer than in colorectal cancer. Consequently, in the context of gastric cancer computational pathology, improved protocols for data exchange are needed.

In the last five years, decentralized machine learning approaches have been proposed which could alleviate the need for physical data exchange. The most prominent examples include federated learning (FL) and swarm learning (SL) [15–17]. In these approaches, multiple datasets are located on physically separate computers, with the DL model trained on each computer separately [16]. In these distributed learning protocols, multiple partners co-train AI models and exchange the learned model parameters at regular intervals during the training process. In this way, information from all training datasets is acquired without ever having access to any data other than the local training dataset. In FL, the model aggregation takes place at a central server, which sends back the merged DL model to all participants. In SL, there is no central server. Instead, all participants communicate with each other on a peer-to-peer level, coordinated by an Ethereum-based blockchain. SL has been successfully employed in experimental use cases in the analysis of transcriptomic data and X-Ray images [16] as well as computational pathology in colorectal cancer [17].

The objective of the present study was to evaluate the feasibility of SL for computational pathology-based biomarker discovery in gastric cancer.

## Methods

### Ethics statement

All experiments were conducted in accordance with the Declaration of Helsinki and the International Ethical Guidelines for Biomedical Research Involving Human Subjects by the Council for International Organizations of Medical Sciences (CIOMS). The collection and analysis of patient samples in each cohort was approved by the Ethics board at each institution as described below.

### Patient cohorts

We collected digital whole-slide images (WSIs) of H&E-stained slides tissue section samples obtained from surgical resections (Table 1). We included four cohorts of patients with gastric cancer from four countries (Switzerland, Germany, the UK and the USA). Three of these cohorts were used as training cohorts and one was used as the testing cohort. Each dataset was stored on a physically separate computer. The training cohorts were BERN (*N* = 417) from the pathology archive at Inselspital, University of Bern (Bern, Switzerland) [18], LEEDS (*N* = 906) from Leeds Teaching Hospital National Health Service Trust (Leeds, United Kingdom) [19], TUM (*N* = 601) samples from Institute of Pathology at the Technical University Munich, Germany [20]. Patients in BERN and LEEDS were not pretreated with neoadjuvant therapy, while approximately half of the patients in the TUM cohort received neoadjuvant therapy [20]. The external validation cohort was the TCGA (*N* = 433) which is a subset of the publicly available data “The Cancer Genome Atlas” from the USA [21].

**Table 1:** Clinico-pathological features of all cohorts

	BERN	LEEDS	TUM	TCGA
Use in this study	Train	Train	Train	Test
Cohort type	Population	Population	Population	Population
*N* Patients in cohort	418	903	601	443
Age (median)	70.94	70.095	64.7	NA
Age (IQR)	8.079	6.859	16.0	NA
Gender: male	258	586	439	285
Gender: female	160	314	162	158
Gender: unknown	0	3	0	0
MSS/pMMR	366	632	544	308
MSI/dMMR	49	70	57	75
Unknown MSI status	3	201	1	60
MSI/MMR method	IHC	IHC	PCR	PCR
EBV status: negative	405	738	577	353
EBV status: positive	11	30	24	30
EBV status: unknown	0	138	1	60
EBV detection method	EBER ISH	EBER ISH	EBER ISH	Genetic test [21]
Stage 1	49	117	53	59
Stage 2	54	94	78	130
Stage 3	150	265	321	183
Stage 4	165	427	149	44
Stage unknown	0	3	0	27
Scanner file format description	Aperio digital Slide	Aperio image library v10.0.50	Aperio image library v12.0.15	Aperio image library vFS90

*EBER ISH* Epstein–Barr encoding region in situ hybridization, *IHC* immunohistochemistry, *NA* not available

### End-to-end prediction workflow

We used a weakly supervised end-to-end prediction workflow for binary classification tasks [1, 3]. “Weakly supervised” in this context means that the target labels are only defined on the level of whole-slide images, but the actual computational analysis is performed on the level of tiles. Our objective was to predict MSI status (MSI vs. microsatellite stable (MSS)) or EBV status (positive vs. negative) directly from image data. We preprocessed the histological WSIs by scanning them on Leica Aperio Scanners at 20× magnification using the “Histology Image Analysis (HIA)” routines [1, 22] according to the “Aachen Protocol for Deep Learning Histopathology”, as described previously [23]. Due to the high resolution of histology WSIs, we tessellated them into non-overlapping tiles of $(512\times 512 \times 3)$ pixels and color-normalized using the Macenko method [24]. During this process, we removed blurry patches as well as non-tissue background from the dataset using canny edge detection [1]. We subsequently resized each patch to $(224\times 224 \times 3)$ and used the pre-trained “RetCCL” convolutional neural network [25, 26] to extract a $(2048\times 1)$ feature vector from 200 randomly selected patches for each patient. This decision was based on previous work demonstrating that 200 patches are sufficient to obtain robust predictions [6]. The feature vectors subsequently served as an input to a fully connected classification network. The classification network consisted of seven layers with (2048 × 2048), (2048 × 1024), (1024 × 512), (512 × 256), (256 × 256), (256 × 128) and (128 × 2) connections with a ReLU activation function. No manual annotations of tumor tissue were used and the image tiles were generated from the full whole-slide image.

### Swarm learning workflow

Swarm learning (SL) enables the co-training of machine learning models across multiple computers at separate physical locations whereby each computer has its own set of proprietary data and no raw data are shared between the computers. In this study, we trained a model in an SL network of three separate computers called “peers”. Model weights were sent from each peer to the other peers on multiple synchronization events (sync events) at the end of each synchronization interval. Thereafter, model weights were averaged at each sync event and training continued at each peer with the averaged parameters. In the SL implementation which we used, metadata about the model synchronization is stored on an Ethereum blockchain. In this setup, the blockchain manages the global status information about the model. Motivated by a previous study in colorectal cancer [17], we used weighted SL as the default approach. This means that the weights contributed by each peer were multiplied with a weighting factor that was proportional to the data which the partner contributed. We used the Hewlett Packard Enterprise (HPE) SL implementation, which consisted of four components: the SL process, the Swarm Network (SN) process, identity management, and HPE license management. All processes (also called nodes in the original HPE implementation) were run in a Docker container. A detailed description of this process with a small sample dataset and instructions on how to reproduce our experiments is available together with our code can be found below.

### Experimental design

We initially trained separate MSI and EBV prediction models on each of the training cohorts individually. Thereafter, all training cohorts were collected on a single computer and a new model was trained on the merged cohort (centralized, or merged cohort). We then trained classifiers using SL, with the SL training process being initiated on three physically separate computers, each containing one of the training cohorts. Finally, all models were externally validated on the test cohort. To examine data efficiency, we repeated all experiments for randomly selected stratified (thus, maintaining class proportions) subgroups of 25, 50, 100, 200 patients per training cohort. MSI and EBV were non-overlapping in our cohorts (which is compatible with previous studies [5]), allowing us to train another set of classifiers for the three-class prediction problem of MSI, EBV-positive and “double-negative” patients. This experiment was performed for the local models, the centralized model, and the SL model.

### Explainability

To investigate the plausibility of model predictions, we used two methods at different scales: whole-slide prediction heatmaps and high-scoring image tiles. Whole-slide prediction heatmaps were generated by visualizing the model prediction as a continuous value with a univariate color map, with gaps linearly interpolated. High-scoring image tiles were generated using the highest-scoring tiles from the highest-scoring patients and checked qualitatively for plausibility by a trainee pathologist (KJH) supervised by a specialty pathologist (HIG). Furthermore, we assessed a possible enrichment of multiple tumor-related properties in misclassified cases compared to all other cases in the test cohort, the TCGA cohort, based on the SL-trained model. For this analysis, misclassified cases (false positives and false negatives) were defined as the 33% of patients with the lowest predicted score for the class of interest. For example, when predicting MSI status, the misclassified cases were the “true MSI” patients with the lowest MSI score. The investigated tumor properties were WHO grading, Laurén classification, and anatomical region within the stomach as well as four tumor microenvironment properties obtained from Thorsson et al. [27] (data available at https://github.com/KatherLab/cancer-metadata/tree/main/tcga): Leukocyte fraction, Stromal fraction, Intratumor heterogeneity and tumor-infiltrating lymphocyte (TIL) regional fraction. To test for significant differences between the cases of interest (COI) and all others (AO), we used the Chi-square test for categorical variables and a two-tailed unpaired *t* test for continuous variables.

### Statistics

All experiments were repeated three times with different random seeds. The primary statistical endpoint was the area under the receiver operating curve (AUROC) for classification performance. The AUROCs of three training runs (technical repetitions with different random starting values) of a given model were compared. A two-sided unpaired t test with *p* < 0.05 was considered statistically significant. No correction for multiple testing was applied. AUROCs are reported as mean ± standard deviation. All computer systems in this study used consumer hardware and were equipped with Nvidia GPUs.

### Data availability

Data from the TCGA archive are available at https://portal.gdc.cancer.gov/projects/TCGA-STAD. All other data are proprietary and belong to their respective centers (BERN cohort to pathology archive, Institute of Pathology, University of Bern; LEEDS cohort to Leeds Teaching Hospital National Health Service Trust and TUM cohort to Institute of Pathology at the Technical University Munich, Germany). All raw experimental results are available in Suppl. Table 1.

### Code availability

All source codes are available at https://github.com/KatherLab/SWARM and are based on and require the HPE implementation of Swarm Learning, which is publicly available at https://github.com/ HewlettPackard/swarm-learning.

## Results

### Prediction of microsatellite instability with deep learning in local models

**Fig. 1:**
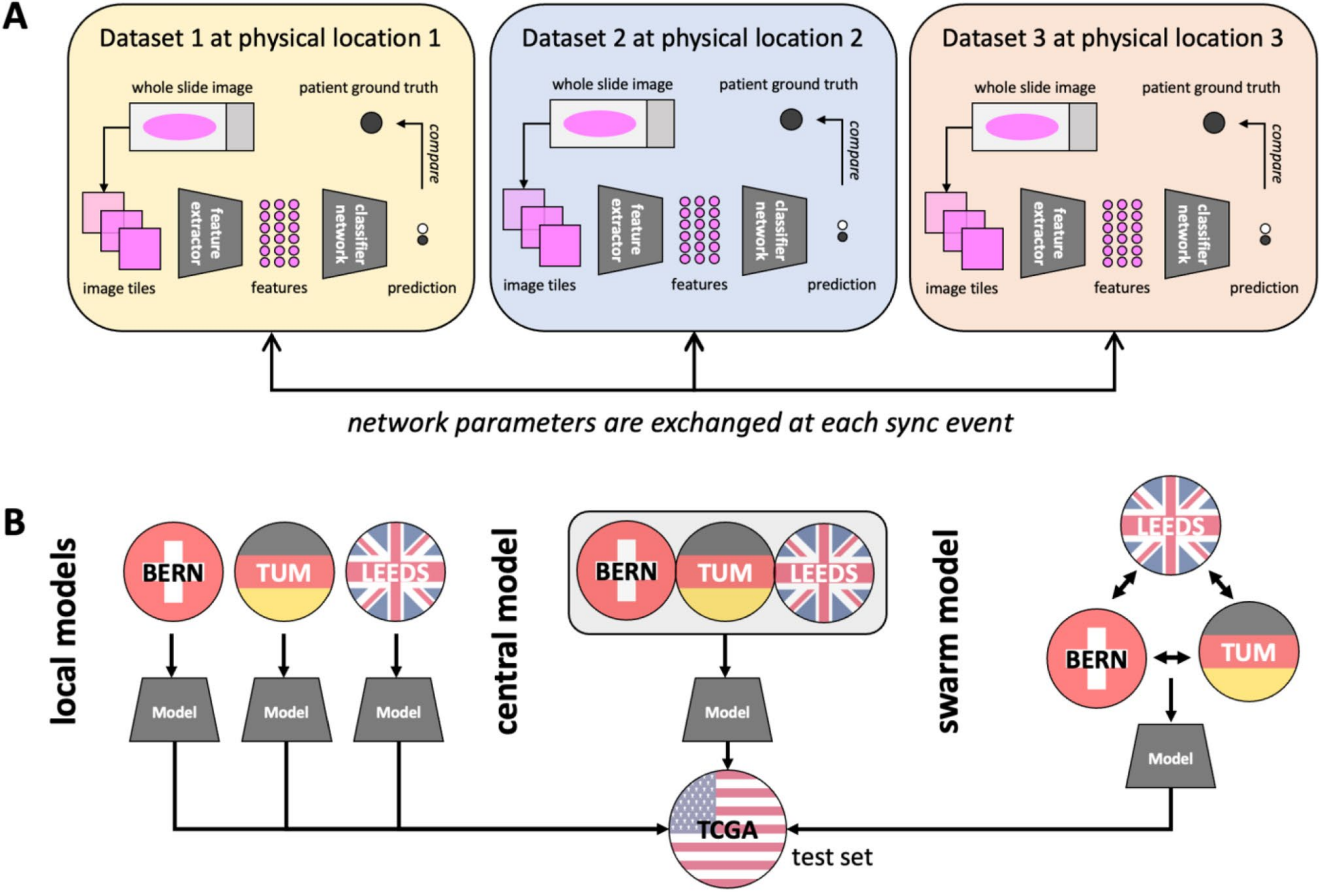
Outline of this study. **A** Technical setup of the swarm learning experiment. **B** Distribution of training and testing set for the three experiments local models (each dataset is used to independently train a model), central models (all datasets are merged), and swarm model (all datasets are used to co-train a model without merging any raw data)

**Fig. 2:**
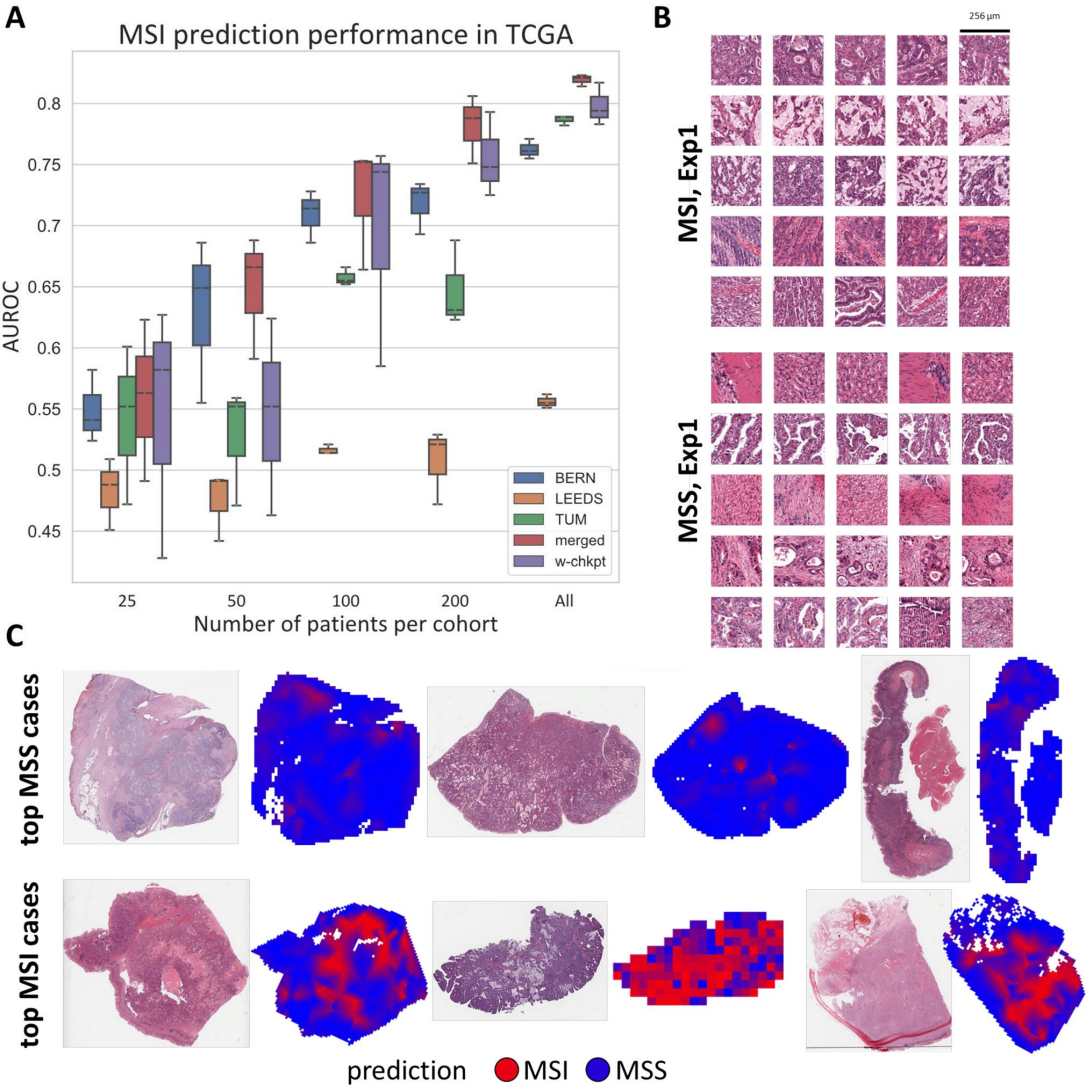
MSI status prediction from pathology images in gastric cancer with swarm learning. **A** Classification performance (area under the receiver operating curve, AUROC) for prediction of MSI status on a patient level in the TCGA cohort. The results of three replicates per experiment are shown as a box plot. The box shows the median and quartiles as the whiskers expand to the rest of the distribution, with the exception of points identified as outliers. **B** Highly predictive image tiles for the Swarm Learning model for MSI and MSS, obtained from the first of three experiments. **C** Whole-slide prediction heatmaps for MSI and MSS in six patients. Abbreviations: *w-chkpt* weighted checkpoint of the swarm (= final swarm learning model), *MSI* microsatellite instable, *MSS* microsatellite stable

In the first experiment, we evaluated the predictability of MSI status directly from pathology images of gastric cancer. We trained independent MSI classifiers on three separate training sets and used the TCGA cohort (*n* = 443) as an external validation set (Fig. 1A, B). The local models showed a highly dataset-dependent performance with AUROCs of 0.7569 (SD ± 0.0034), 0.5583 (SD ± 0.0063) and 0.7843 (SD ± 0.0040) when trained on the BERN (*N* = 418 patients), LEEDS (*N* = 903 patients) and TUM (*N* = 602 patients) cohorts, respectively (Fig. 2A). When the training data were restricted to only a subset of patients in each training cohort, the performance decreased considerably. When the training cohort was limited to 25 patients per cohort, all three local models achieved essentially a random performance with AUROCs of 0.5484 (± 0.0298), 0.4820 (± 0.0293), and 0.5389 (± 0.0660) for models trained on BERN, LEEDS, and TUM, respectively (Fig. 2A). For 50 patients per cohort, only the BERN model reached a non-random performance with an AUROC of 0.6275 (± 0.0675). In general, for any patient number below 100 per cohort, local models had a rather low and highly variable performance with a pronounced variability in performance between multiple experimental repetitions.

### Prediction of microsatellite instability with deep learning in centralized and swarm models

To assess the highest possible performance that can be achieved using our present datasets, we collected the cohorts BERN, LEEDS and TUM on a single computer, trained a centralized MSI classifier on the merged dataset and validated the classifier on the TCGA cohort (Table 2). Training on this larger multicentric dataset consistently improved the performance on the validation set, resulting in an AUROC of 0.8199 (SD ± 0.0051). When reducing the number of training patients per cohort, this performance remained stable for 200 patients per cohort (AUROC of 0.7813 ± 0.0280) and 100 patients per cohort (AUROC of 0.7217 ± 0.0510), but markedly degraded to an AUROC of below 0.65 for any lower patient number (Fig. 2A). The performance of the centrally trained models likely represents an upper limit of the performance that can be reached with our prediction algorithm on the given data. We then assessed the performance of the swarm-trained models in a similar fashion and found that the performance was comparable to the centralized model. For the SL model trained on all data, the AUROC on the test set was 0.8092 (± 0.0132), which was not significantly different from the centralized models (*p* = 0.2648 for swarm vs. merged dataset). Similarly, when the number of patients was restricted to 200 per cohort, the AUROC on the test set was 0.7548 (± 0.0345), which was not statistically significantly different from the centralized models (*p* = 0.3635).

**Table 2:** Prediction performance of MSI prediction, and significance compared to the SL approach

	*N* = 25 patients	*N* = 50 patients	*N* = 100 patients	*N* = 200 patients	All patients
Trained on BERN only	AUROC: 0.5484 (± 0.0298)	AUROC: 0.6275 (± 0.0675)	AUROC: 0.7091 (± 0.0213)	AUROC: 0.7177 (± 0.0219)	AUROC: 0.7569 (± 0.0034)
	*p* val: 0.9601	*p* val: 0.2403	*p* val: 0.8169	*p* val: 0.1895	*p* val: 0.0027
Trained on LEEDS only	AUROC: 0.4820 (± 0.0293)	AUROC: 0.4744 (± 0.0285)	AUROC: 0.5163 (± 0.0040)	AUROC: 0.5066 (± 0.0308)	AUROC: 0.5583 (± 0.0063)
	*p* val: 0.3711	*p* val: 0.2222	*p* val: 0.0318	*p* val: 0.0007	*p* val: 7.8E-06
Trained on TUM only	AUROC: 0.5389 (± 0.0651)	AUROC: 0.5257 (± 0.0489)	AUROC: 0.6576 (± 0.0073)	AUROC: 0.6466 (± 0.0354)	AUROC: 0.7843 (± 0.0040)
	*p* val: 0.9577	*p* val: 0.7447	*p* val: 0.5342	*p* val: 0.0194	*p* val: 0.0355
Trained on all (merged)	AUROC: 0.5563 (± 0.0660)	AUROC: 0.6469 (± 0.0508)	AUROC: 0.7217 (± 0.0510)	AUROC:0.7813 (± 0.0280)	AUROC: 0.8199 (± 0.0051)
	*p* val: 0.8607	*p* val: 0.1375	*p* val: 0.6817	*p* val: 0.3635	*p* val: 0.2648
Trained on all (SL)	AUROC: 0.5385 (± 0.1043)	AUROC: 0.5422 (± 0.0806)	AUROC: 0.6906 (± 0.0957)	AUROC: 0.7548 (± 0.0345)	AUROC: 0.8091 (± 0.0132)

*p* values represent the comparison to swarm learning (corresponding column in the bottom row) with a two-tailed, unpaired *t* test without correction for multiple testing

### Explainability of the swarm-trained model

Next, we investigated if the swarm-trained models detect plausible morphological patterns which are associated with the molecular class of interest. We visualized the highest-scoring image tiles for all class predictions in the TCGA dataset, using the swarm model (Fig. 2B). We found that a number of the MSI tiles with high scores assigned by the model exhibited diverse morphological patterns which are consistent with previously described patterns of MSI gastric cancer [28] (Fig. 2B, Suppl. Fig. 5). MSS tiles, however, contained tissue that was more varied and included tumor but also non-tumor tissue, indicating that the model might have learned that an absence of MSI-specific patterns indicates MSS (Fig. 2B, Suppl. Fig. 6). We then analyzed the whole-slide heat maps for MSS and MSI cases and found that true MSS cases were spatially homogeneously predicted to be MSS, while true MSI cases had large contiguous areas of MSI-predicted areas, allowing the model to make the prediction of MSI at a slide level (Fig. 2C). This shows that the tile-wise processing of whole-slide images of gastric cancer in a swarm learning setup is justified. To further investigate the predictions made by the model, we analyzed the distribution of histopathological features in misclassified cases (Suppl. Fig. 7, 8, 9, 10). We found that cases which were wrongly classified as MSI by the model had significantly (*p* = 0.0089, Suppl. Fig. 7) higher scores for intratumor heterogeneity as defined by Thorsson et al. [27]. Cases which were wrongly classified as MSS by the model had a significantly lower Leukocyte fraction score (*p* = 0.0316, Suppl. Fig. 8), indicating that a paucity of inflammatory cells in the tissue makes the model more likely to classify a case as MSS.

### Prediction of Epstein–Barr virus presence with swarm learning

**Fig. 3:**
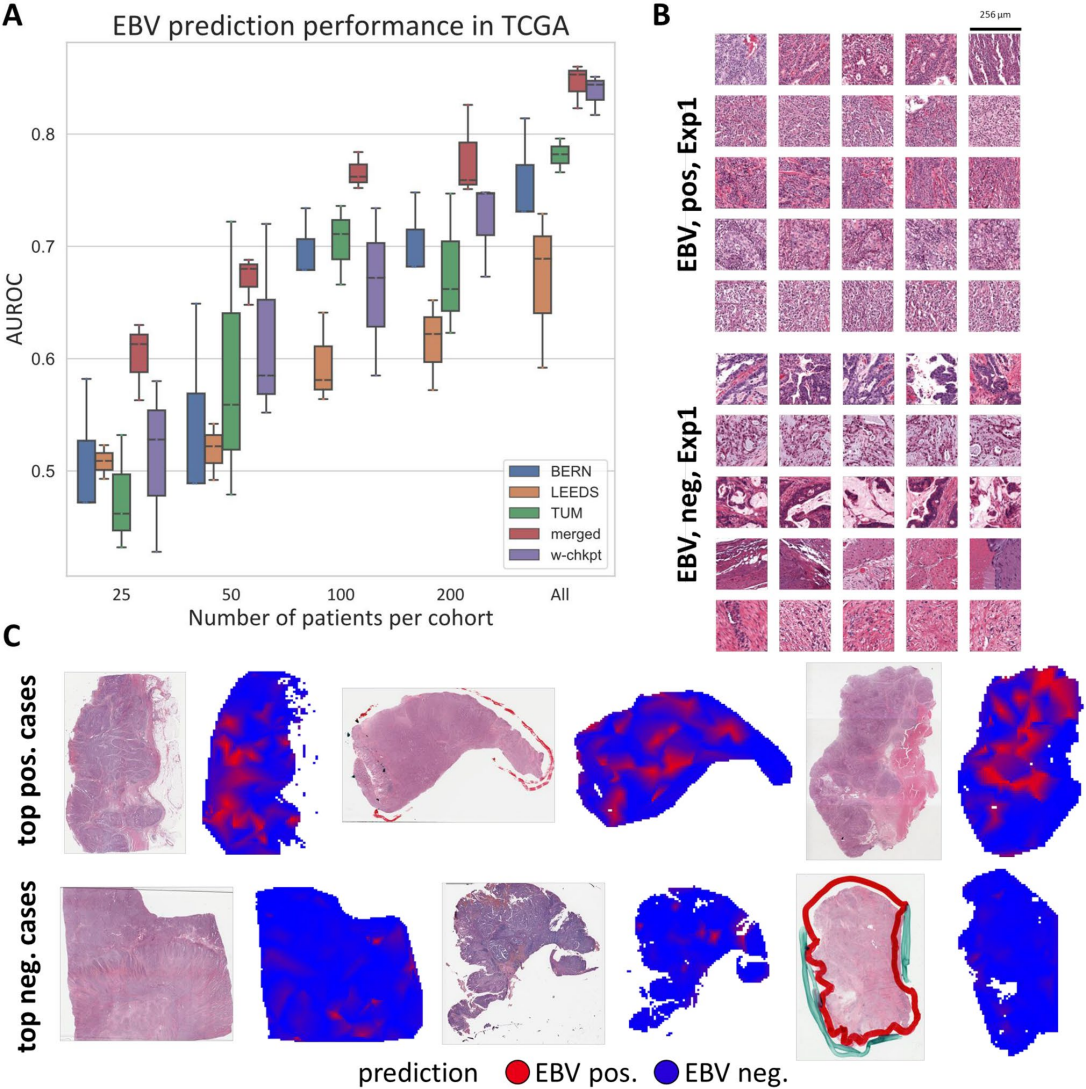
EBV status prediction from pathology images in gastric cancer with swarm learning. **A** Classification performance (area under the receiver operating curve, AUROC) for prediction of EBV status on a patient level in the TCGA cohort. The results of three replicates per experiment are shown as a box plot, obtained from the first of three experiments. The box shows the median and quartiles as the whiskers expand to the rest of the distribution, with the exception of points identified as outliers. **B** Highly predictive image tiles for the Swarm Learning model for MSI and MSS. **C** Whole-slide prediction heatmaps for EBV positivity and negativity in six patients. Abbreviations: *w-chkpt* weighted checkpoint of the swarm (= final swarm learning model), *EBV* Epstein–Barr Virus, *Pos*. positive, *Neg*. negative

To validate our methodology of SL-based biomarker predictability from pathology slides, we addressed another clinically relevant prediction task in the same experimental setup, namely the presence of Epstein–Barr virus RNA in gastric cancer tissue (Table 3). We evaluated the patient-level performance for the prediction of EBV status in the TCGA cohort (*N* = 383 patients, Fig. 3A). We found that models trained on local data achieved AUROCs of 0.7576 (± 0.0479), 0.6674 (± 0.0704) and 0.7812 (± 0.01501) when trained on BERN, LEEDS and TUM, respectively. Similar to MSI prediction, merging the three training cohorts on a central computer improved the performance to an AUROC of 0.8451 (± 0.0196). This was compared to the performance of SL-trained models, which achieved an AUROC of 0.8372 (± 0.0179). Like in MSI prediction, this performance was also not significantly (*p* = 0.6301) different from the performance of the centrally trained model. In this task, however, the swarm-trained model was somewhat less data efficient than the centrally trained model when trained on only a subset of all patients in each cohort (Fig. 3A). We then investigated the explainability of the swarm model-based predictions. First, we investigated properties of misclassified cases. Cases which were misclassified as EBV positive had a significantly higher tumor-infiltrating lymphocyte score [27] compared to the rest of the cohort (*p* < 0.0001, Suppl. Fig. 9), indicating that a higher lymphocytic infiltration makes the model more likely to call the case “EBV positive”. No significant associations were observed for false negatives, i.e., cases which were misclassified as EBV negative (Suppl. Fig. 10). In addition, we visually assessed highly scoring image tiles as predicted by the model. EBV-positive tiles tended to contain more poorly differentiated tumor (Fig. 3B, Suppl. Fig. 11) than tiles predicted to be EBV negative (Fig. 3B, Suppl. Fig. 12). In the prediction heatmaps for whole slides (Fig. 3C), EBV-positive cases had contiguous regions of predicted EBV positivity, while EBV-negative cases were almost completely predicted to be EBV negative by the model (Fig. 3C). In addition, we observed that the deep learning procedure was not obviously affected by the presence of pen marks in the TCGA test set (Fig. 3B). Because EBV and MSI were non-overlapping in our cohorts, we also trained a model on the three-class problem (EBV–MSI–double negative). We found that this approach gave comparable results: The centralized and the SL model were able to predict EBV with an AUROC of above 0.85, MSI with an AUROC of above 0.70 and double negatives with an AUROC of above 0.74 (Suppl. Fig. 13). We conclude that swarm-trained models can yield a high prediction accuracy in prediction of molecular biomarkers gastric cancer, but the robustness can vary between different biomarkers.

**Table 3:** Prediction performance of EBV prediction, and significance compared to the SL approach

	*N* = 25 patients	*N* = 50 patients	*N* = 100 patients	*N* = 200 patients	All patients
Trained on BERN only	AUROC: 0.5061 (± 0.0635)	AUROC: 0.5373 (± 0.0923)	AUROC: 0.6968 (± 0.0317)	AUROC: 0.7033 (± 0.0381)	AUROC: 0.7576 (± 0.0479)
	*p* val: 0.9567	*p* val: 0.3590	*p* val: 0.5129	*p* val: 0.6037	*p* val: 0.0562
Trained on LEEDS only	AUROC: 0.508 (± 0.0150)	AUROC: 0.5182 (± 0.0251)	AUROC: 0.5944 (± 0.0404)	AUROC: 0.6144 (± 0.0404)	AUROC: 0.6674 (± 0.0704)
	*p* val: 0.9396	*p* val: 0.1334	*p* val: 0.2365	*p* val: 0.0345	*p* val: 0.0163
Trained on TUM only	AUROC: 0.4735 (± 0.0513)	AUROC: 0.5782 (± 0.1238)	AUROC: 0.7037 (± 0.0354)	AUROC: 0.6753 (± 0.0634)	AUROC: 0.7812 (± 0.0150)
	*p* val: 0.5311	*p* val: 0.7320	*p* val: 0.4430	*p* val: 0.3633	*p* val: 0.0143
Trained on all (merged)	AUROC: 0.6013 (± 0.0348)	AUROC: 0.6717 (± 0.0211)	AUROC: 0.7658 (± 0.0163)	AUROC: 0.7779 (± 0.0411)	AUROC: 0.8451 (± 0.01965)
	*p* val: 0.1396	*p* val: 0.3724	*p* val: 0.0817	*p* val: 0.1787	*p* val: 0.6301
Trained on all (SL)	AUROC: 0.5079 (± 0.0772)	AUROC: 0.6149 (± 0.0890)	AUROC: 0.6608 (± 0.0748)	AUROC: 0.7217 (± 0.0219)	AUROC: 0.8372 (± 0.0179)

*p* values represent the comparison to swarm learning (corresponding column in the bottom row) with a two-tailed, unpaired *t* test without correction for multiple testing

## Discussion

Computational pathology problems in gastric cancer require large datasets to compensate for the intra- and inter-patient heterogeneity. Preferably, such data should come from different medical centers to avoid bias and achieve models with diverse, generalizable knowledge. However, the collection of such datasets encounters practical, ethical and legal obstacles. Many of these obstacles could be overcome with SL, which enables multiple institutions to collaborate without revealing sensitive patient data.

In this study, we empirically demonstrate that SL is feasible in the context of gastric cancer. We show that prediction of MSI and EBV status from H&E pathology slides with SL yields highly performing classifiers. Prediction of these biomarkers is important as MSI status defines an important clinical subgroup of gastric cancer patients with improved prognosis, and both MSI and EBV status indicate patients that are more likely to respond to immunotherapy than other patients [29]. We observe differences between the two biomarkers: For EBV, the classification problem is more unbalanced. In our training cohort, there were 3.64% EBV-positive cases overall, compared to 10.24% MSI cases overall, which is representative of other cohorts [29]. This represents a challenge for DL as limited case numbers and subsequently images can create difficulty for the algorithm when learning features. This means that not just large datasets are required, but also datasets containing a sufficient quantity of the various desired classifications among the samples, so as to ensure that features pertinent to all classifications (e.g., MSI vs. non-MSI) within the target category (e.g., MSI status) may be accurately learnt by the algorithm. SL, through its decentralized nature and compartmentalisation of patient data, may serve to ease the acquisition of these large and varied datasets by creating fewer barriers in data sharing between institutions, although it does not solve the data imbalance issue.

From a practical point of view, SL could be an alternative in the future to share patient-related data across locations. Regarding the implementation of SL, there are several software frameworks that either offer swarm learning as a commercial product (HPE) or provide open source functionality that could be modified to be used in a SL setup (Nvidia Flare via https://github.com/NVIDIA/NVFlare and Syft by OpenMined via https://github.com/OpenMined/PySyft). None of these frameworks provide easy plug and play functionality yet and setting them up requires considerable expertise in the administration of computers. Making these frameworks more accessible to the less tech-savvy user could facilitate and accelerate their adoption and use in a clinical context.

A limitation of our study is the somewhat unbalanced label classifications in our cohorts. In addition to this, our methodology has only been tested on a small number of biomarkers. It will be important to validate our findings on a greater number of biomarkers in future studies, and in particular clinically relevant biomarkers. Larger cohorts with either a greater number of patients and/or increased number of images per patient could have provided more information for training and ultimately classification. Similarly, data from non-European centers would provide more diverse information, which could improve predictions and generalizability of our model. Another limitation is the limited interpretability of the models. We visualize the highly relevant image tiles, which represent the “typical” morphology for any particular class, as learned by the model. In general, a better understanding of the inner workings of deep learning models would be desirable for this and other biomarker studies in computational pathology. In the future, attention-based DL methods could further improve performance and interpretability [26, 30, 31].

In conclusion, our study demonstrates for the first time the feasibility and benefit of SL for the development of DL-based biomarkers in gastric cancer and demonstrates some obstacles which need to be overcome before a more widespread use of this technology.

## Supplementary Information

Below is the link to the electronic supplementary material.

Supplementary file1 (DOCX 21827 KB)

Supplementary file2 (XLSX 19 KB)
