# Supplementary material for: Direct prediction of genetic aberrations from pathology images in gastric cancer with swarm learning
Source: Gastric Cancer. 2022 Oct 20;26(2):264–74. doi: 10.1007/s10120-022-01347-0 (PMC9950158; doi:10.1007/s10120-022-01347-0)
Supplement: Supplementary file 1 — Supplementary file1 (DOCX 21827 KB) [file 10120_2022_1347_MOESM1_ESM.docx]

# Supplementary Figures


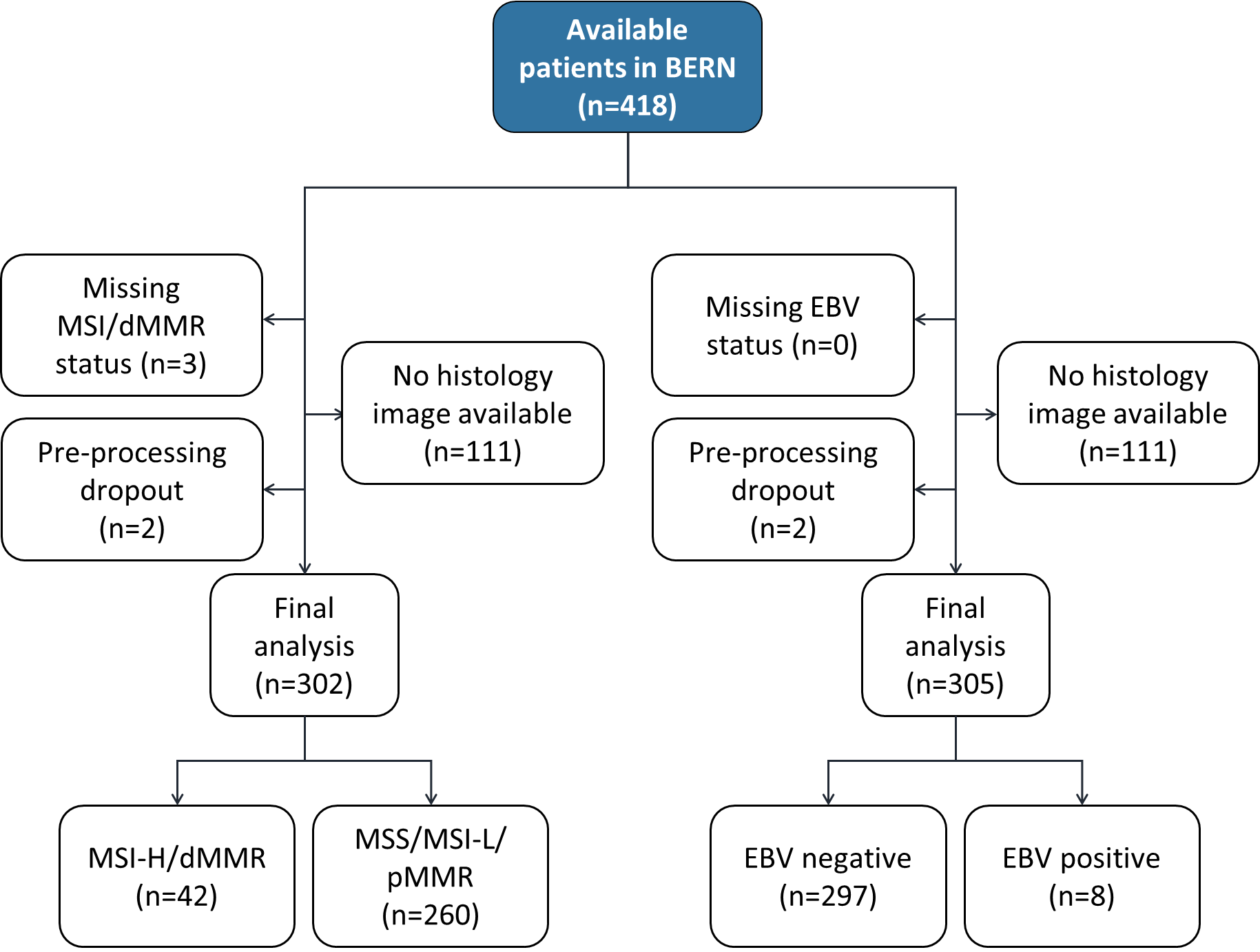


**Suppl. Figure 1: Patient flowchart for the BERN cohort.** This cohort was previously investigated in Muti et al.[13] This cohort was one of the training sets in our study.


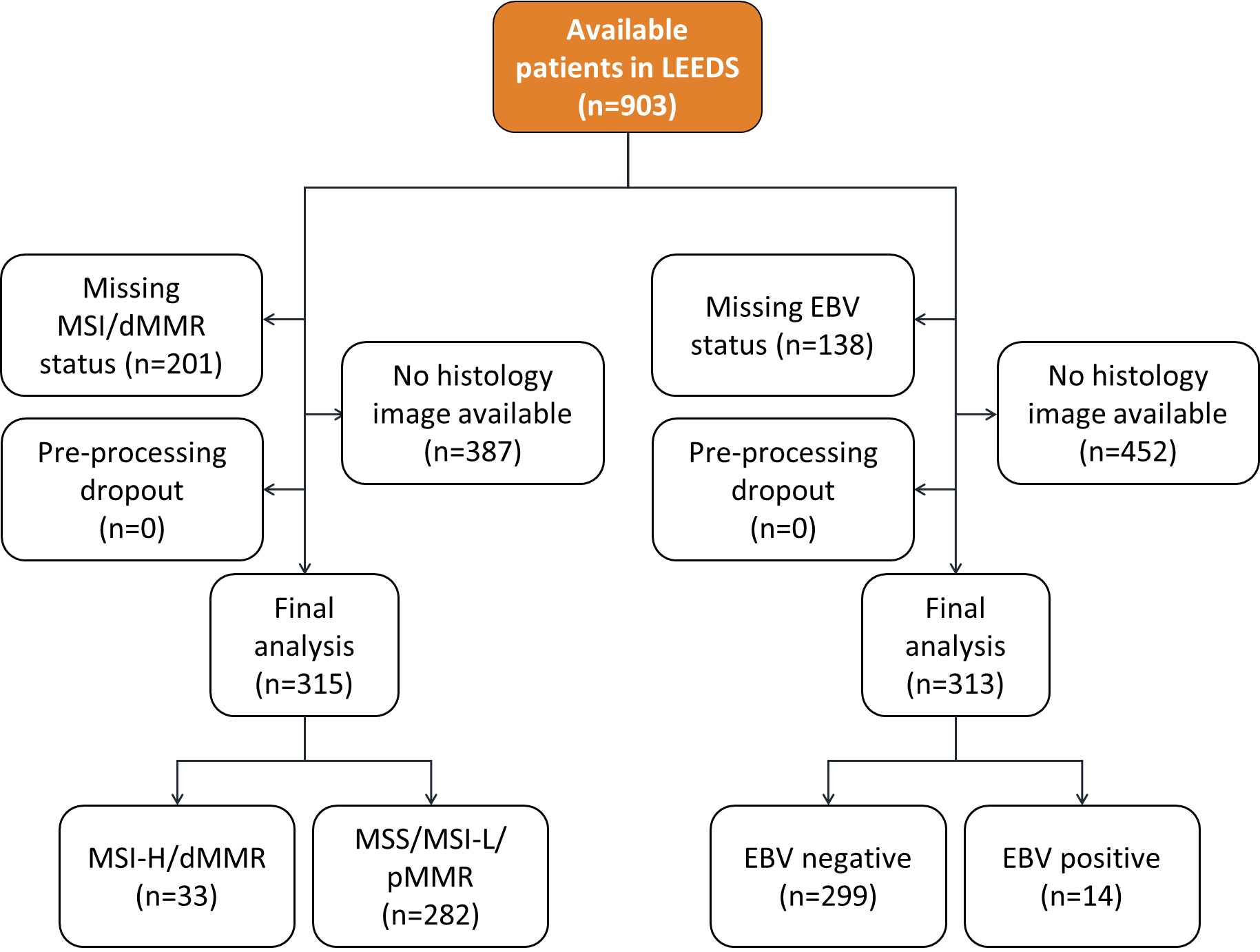


**Suppl. Figure 2: Patient flowchart for the LEEDS cohort.** This cohort was previously investigated in Muti et al.[13] This cohort was one of the training sets in our study.


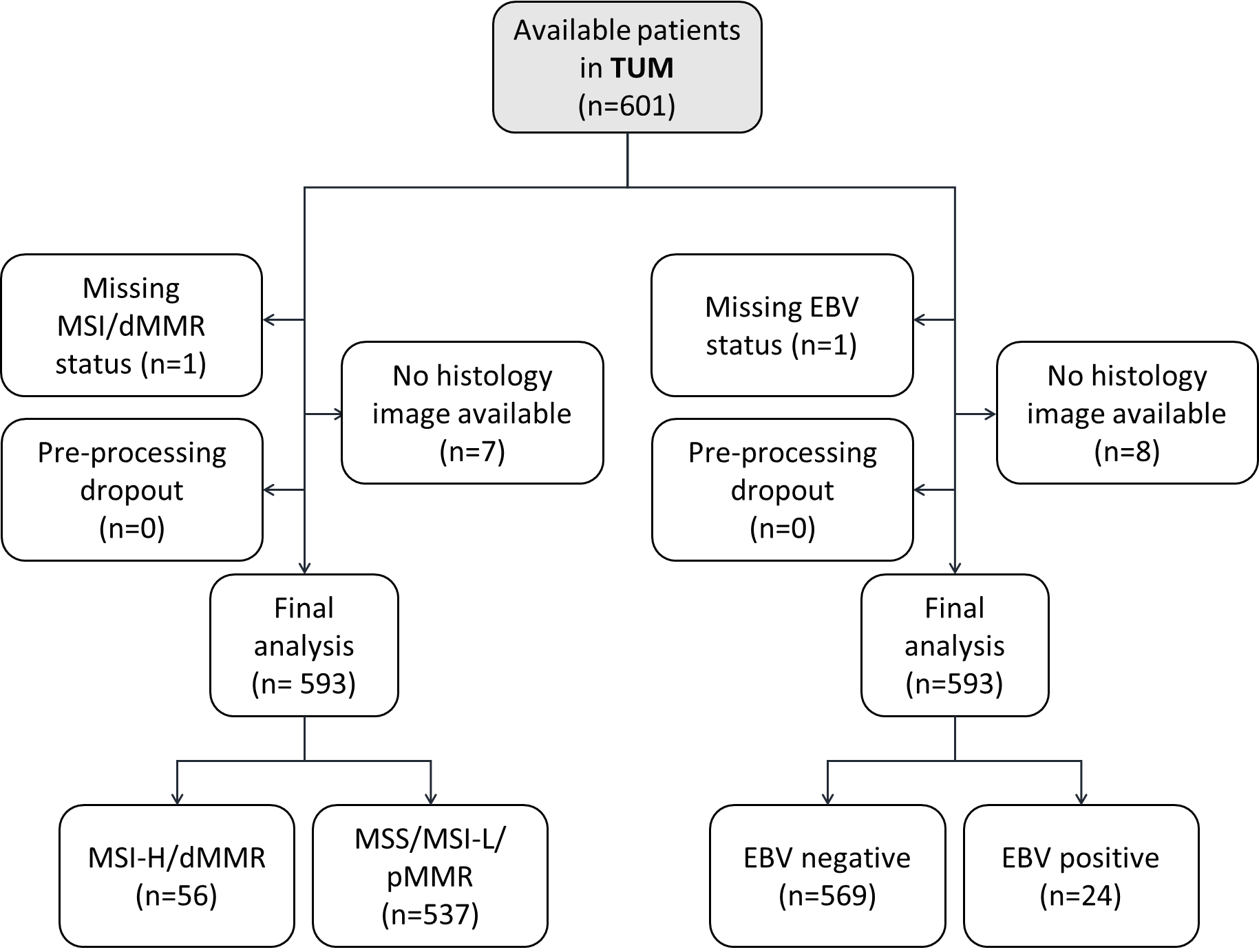


**Suppl. Figure 3: Patient flowchart for the TUM cohort.** This cohort includes the samples used by Muti et al.[13], but includes additional samples and is therefore much larger. This cohort was one of the training sets in our study.


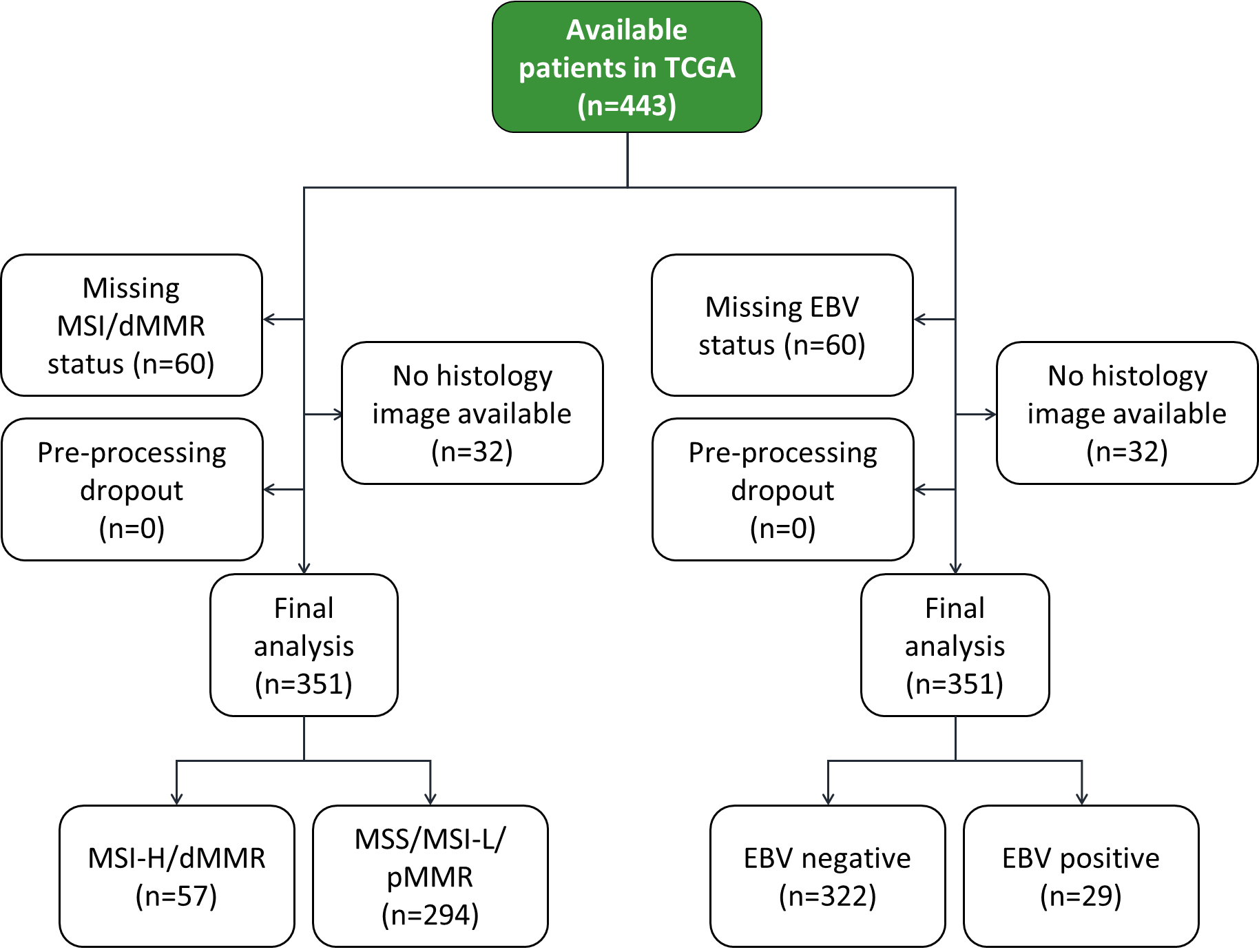


**Suppl. Figure 4: Patient flowchart for the TCGA cohort.** This cohort was previously investigated in Muti et al.[13]. This cohort was used as an external validation (test) set.


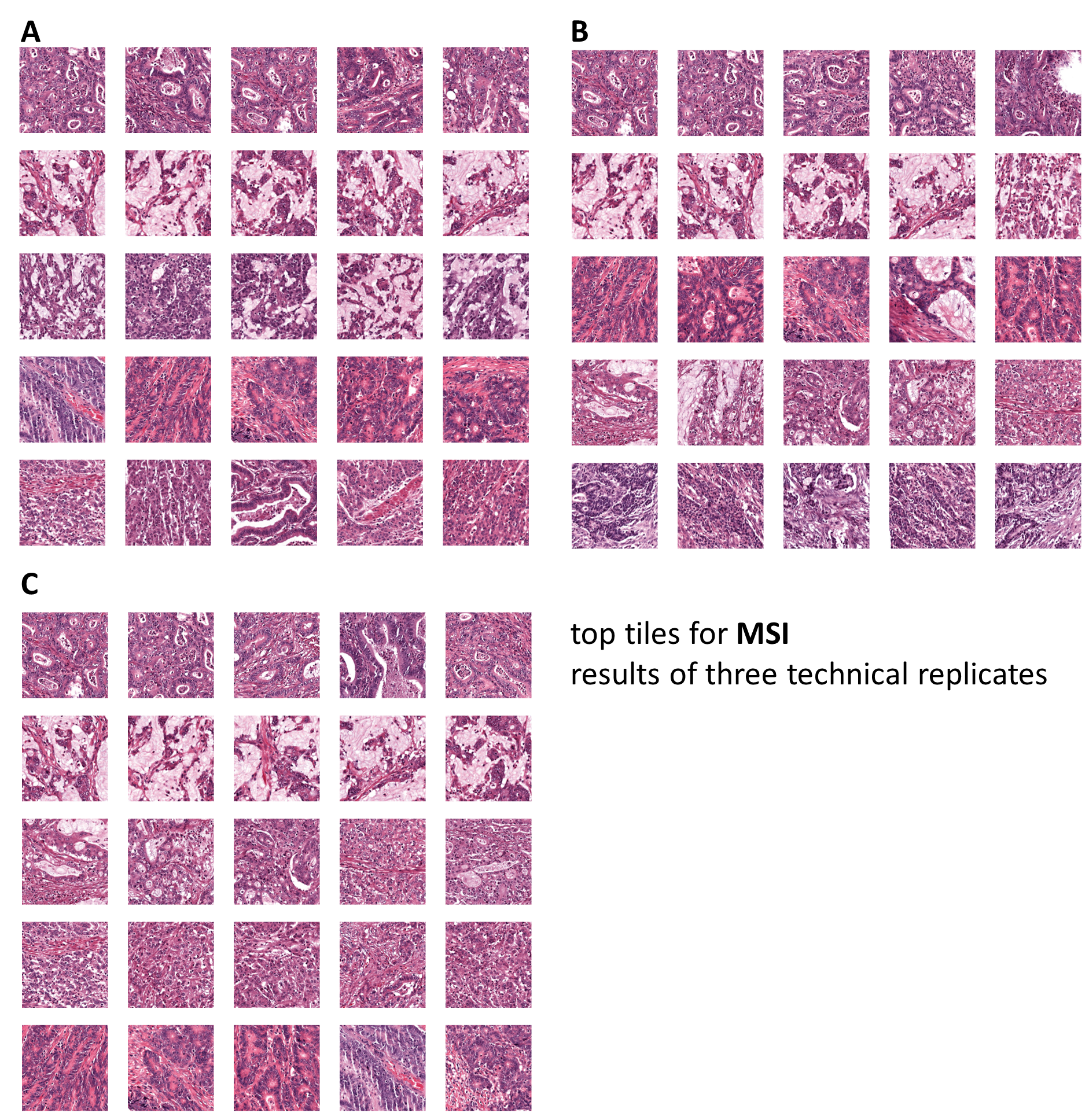


**Suppl. Figure 5: Top tiles for top five predicted patients with MSI, three technical replicates. (A)** Replicate 1, **(B)** Replicate 2, **(C)** Replicate 3.


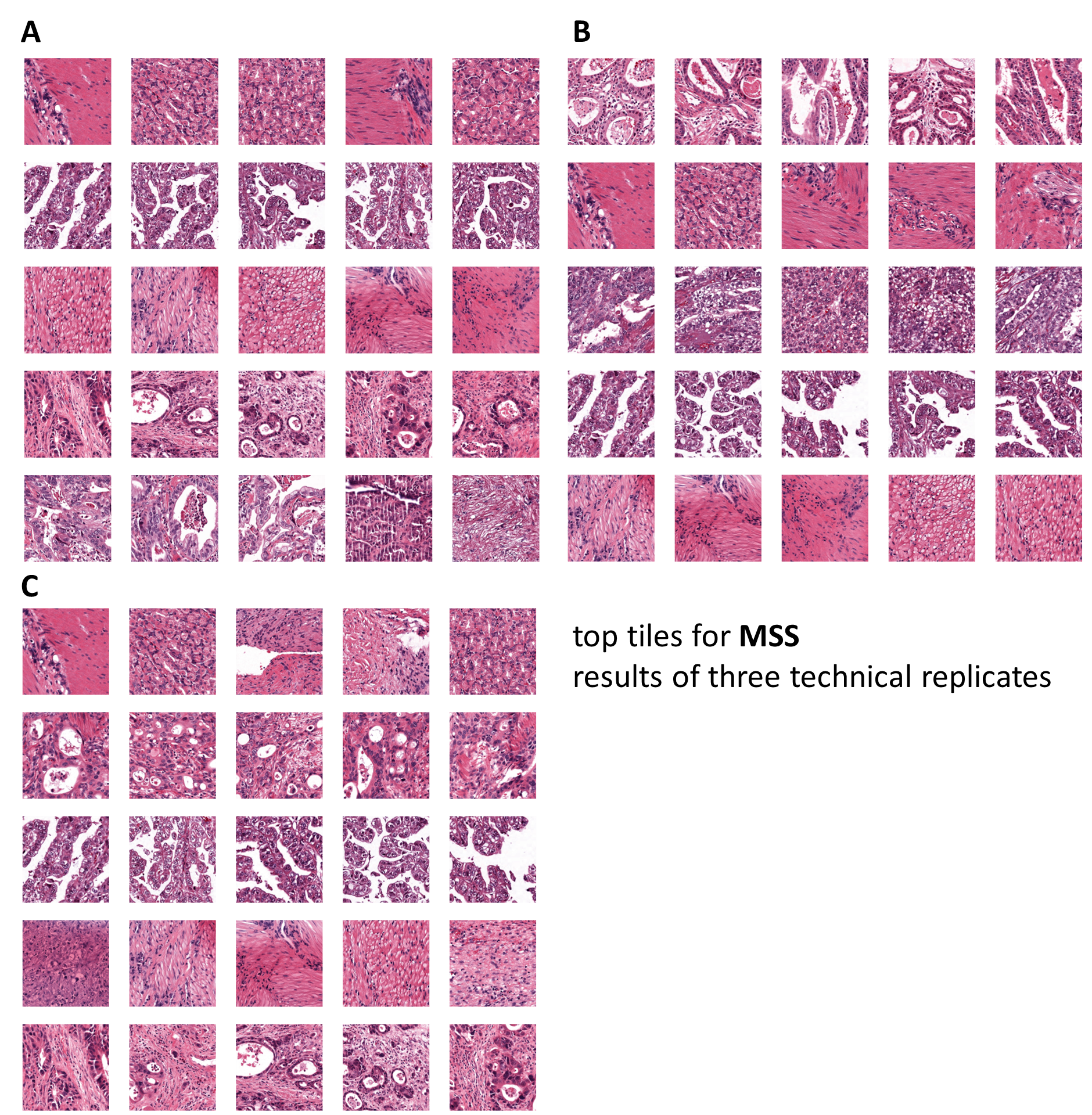


**Suppl. Figure 6: Top tiles for top five predicted patients with MSS, three technical replicates. (A)** Replicate 1, **(B)** Replicate 2, **(C)** Replicate 3.


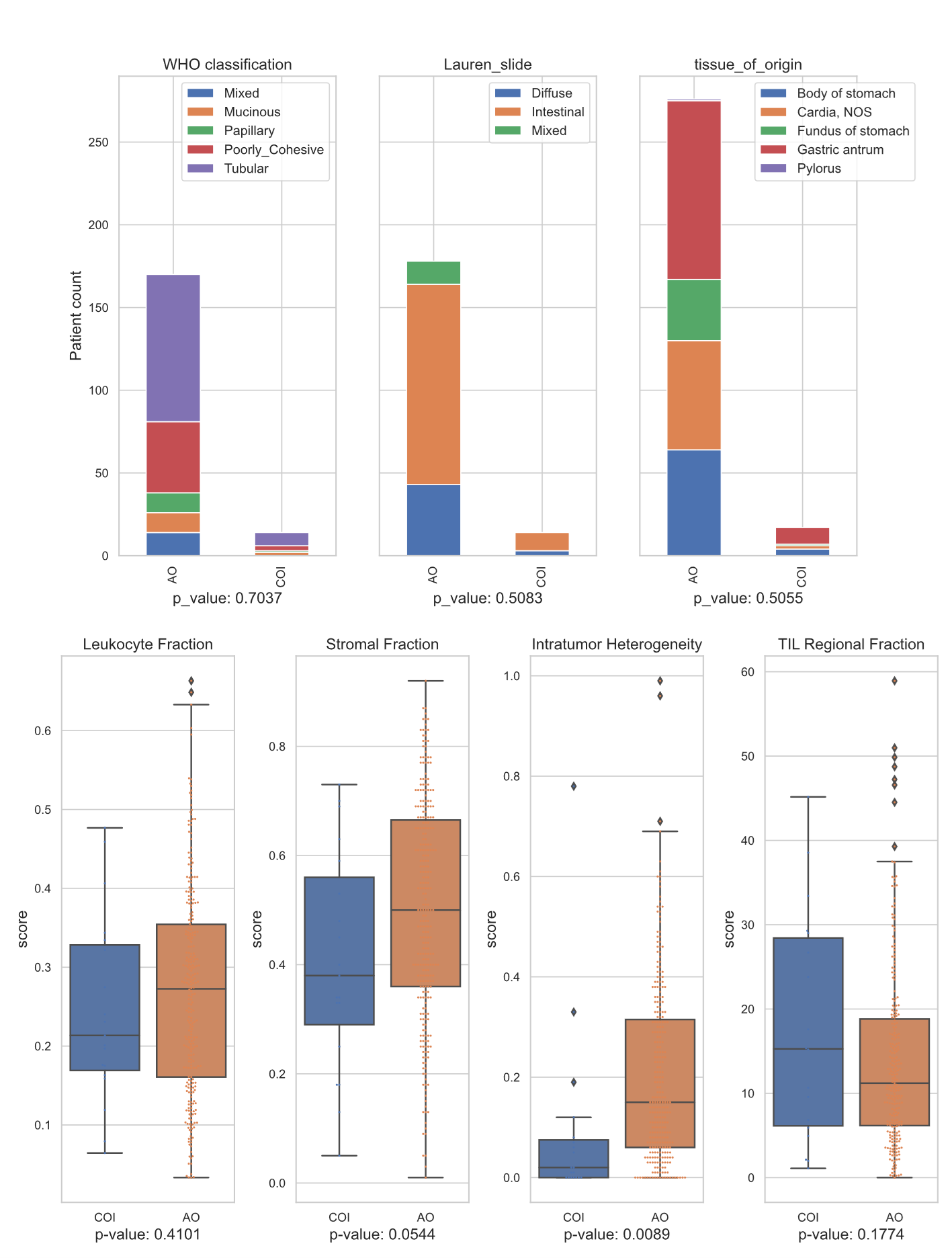


**Suppl. Figure 7: Evaluation of false positive cases for MSI status prediction in the TCGA cohort. COI = class of interest (true MSS, predicted MSI), AO = all others.** Chi-square test of independence was used to obtain the p-value for categorical variables. The two tailed unpaired t-test was used for the continuous variables.


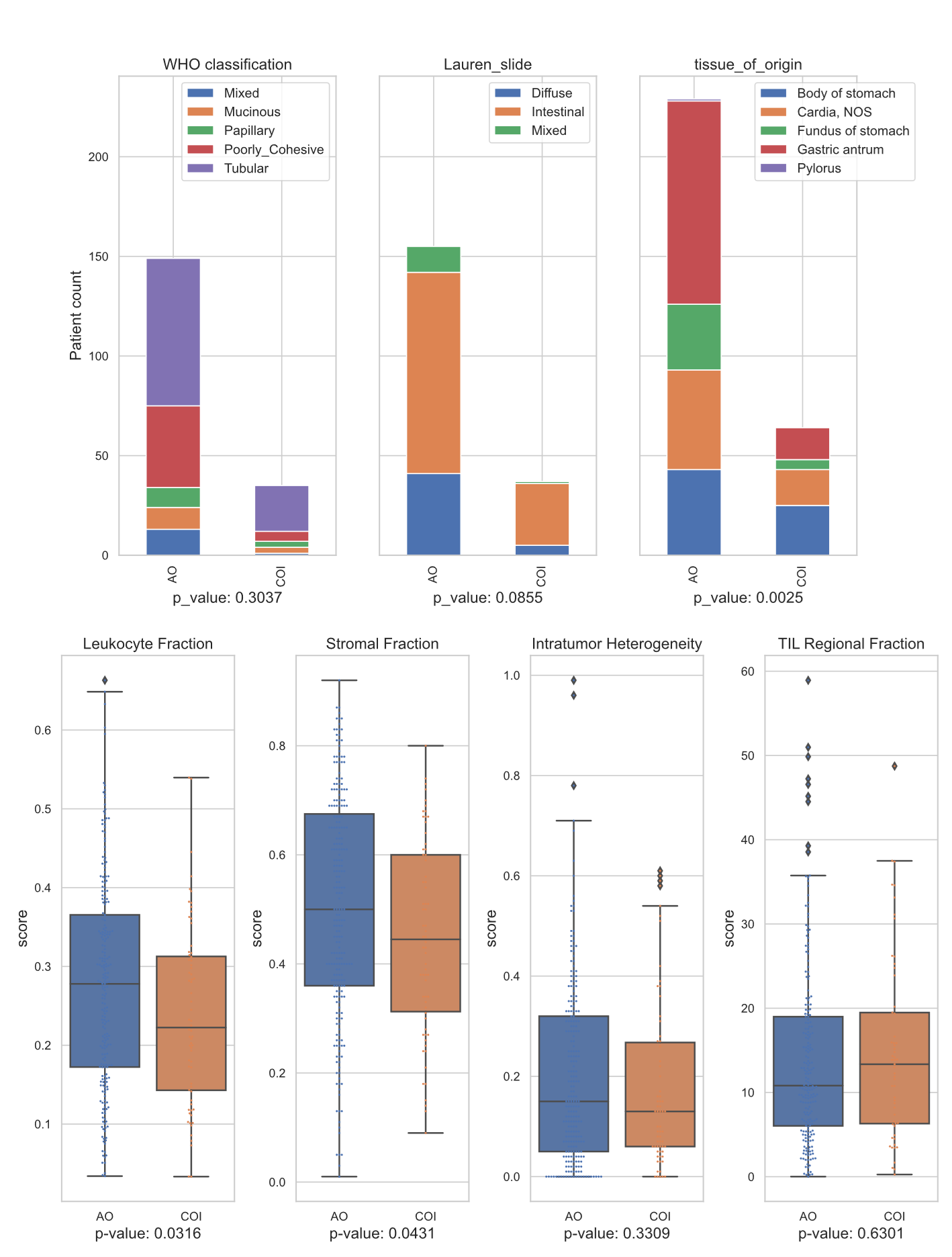


**Suppl. Figure 8: Evaluation of false negative cases for MSI status prediction in the TCGA cohort. COI = class of interest (true MSI, predicted MSS), AO = all others.** Chi-square test of independence was used to obtain the p-value for categorical variables. The two tailed unpaired t-test was used for the continuous variables.


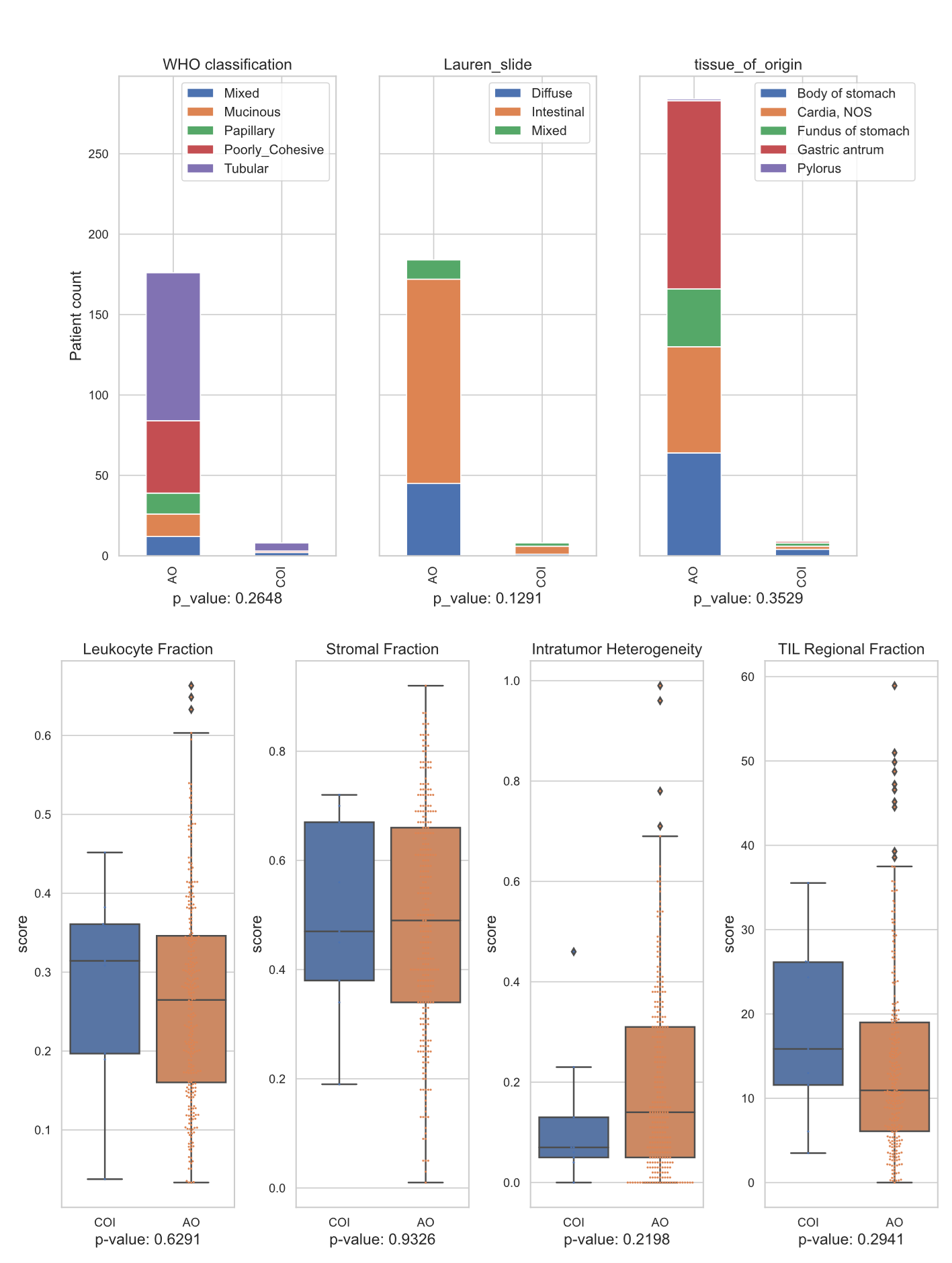


**Suppl. Figure 9: Evaluation of false positive cases for EBV status prediction in the TCGA cohort. COI = class of interest (true EBV negative, predicted EBV positive), AO = all others.** Chi-square test of independence was used to obtain the p-value for categorical variables. The two tailed unpaired t-test was used for the continuous variables. The model for class predictions was the w-chkpt swarm model.


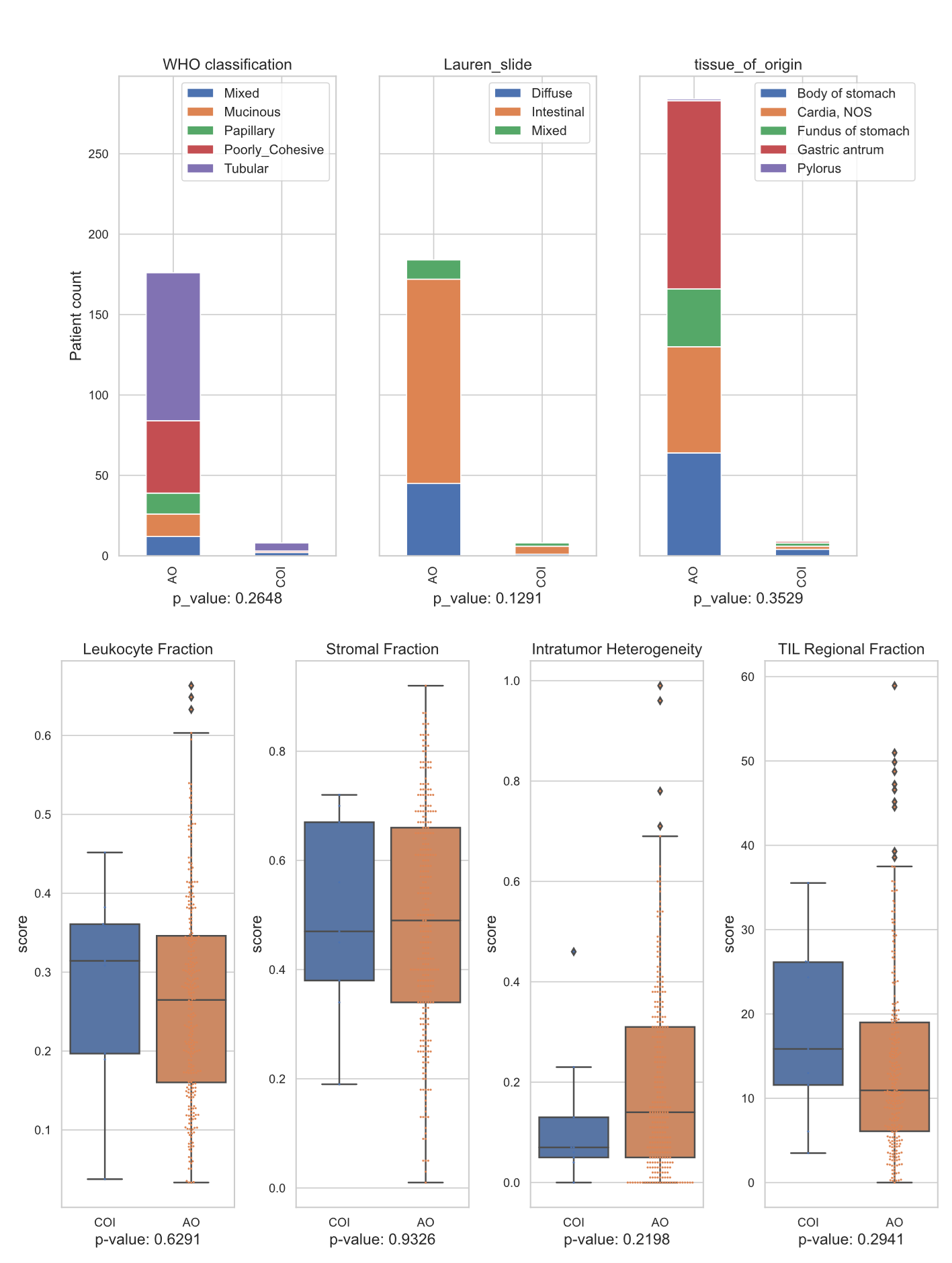


**Suppl. Figure 10: Evaluation of false negative cases for EBV status prediction in the TCGA cohort. COI = class of interest (true EBV positive, predicted EBV negative), AO = all others.** Chi-square test of independence was used to obtain the p-value for categorical variables. The two tailed unpaired t-test was used for the continuous variables. The model for class predictions was the w-chkpt swarm model.

**
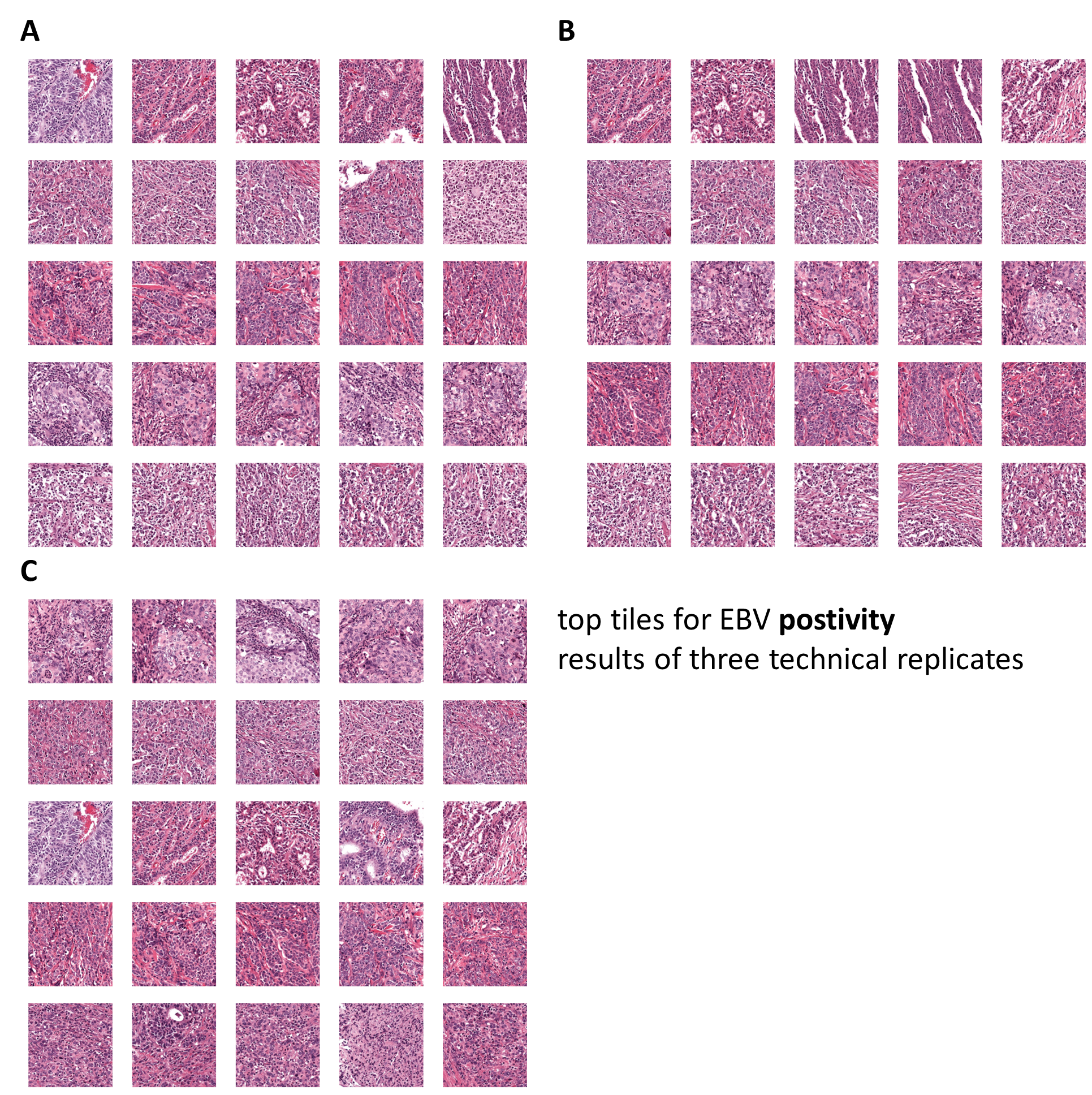
**

**Suppl. Figure 11: Top tiles for top five predicted patients with EBV positivity, three technical replicates. (A)** Replicate 1, **(B)** Replicate 2, **(C)** Replicate 3.

**
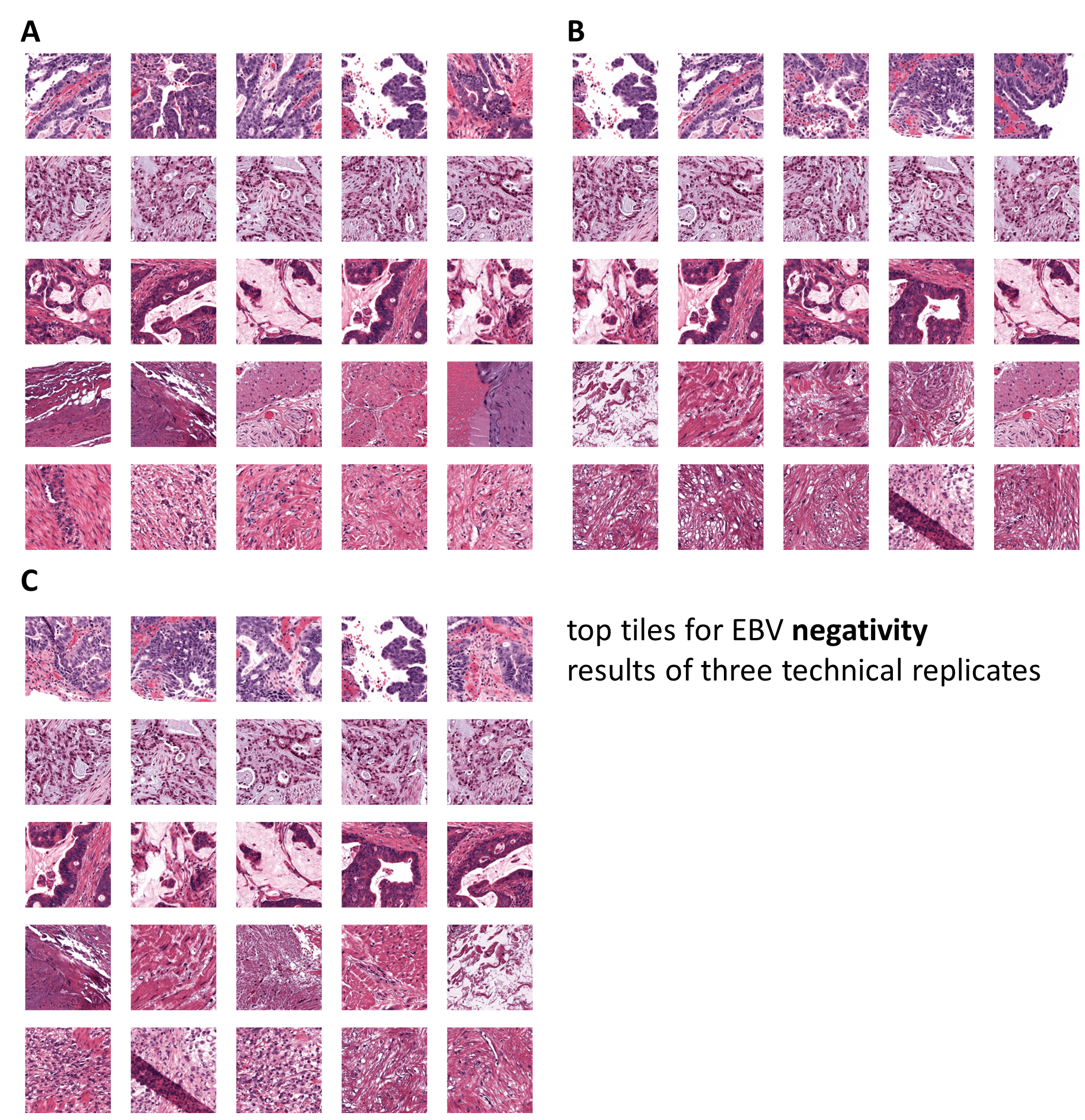
**

**Suppl. Figure 12: Top tiles for top five predicted patients with EBV negativity, three technical replicates. (A)** Replicate 1, **(B)** Replicate 2, **(C)** Replicate 3.

**
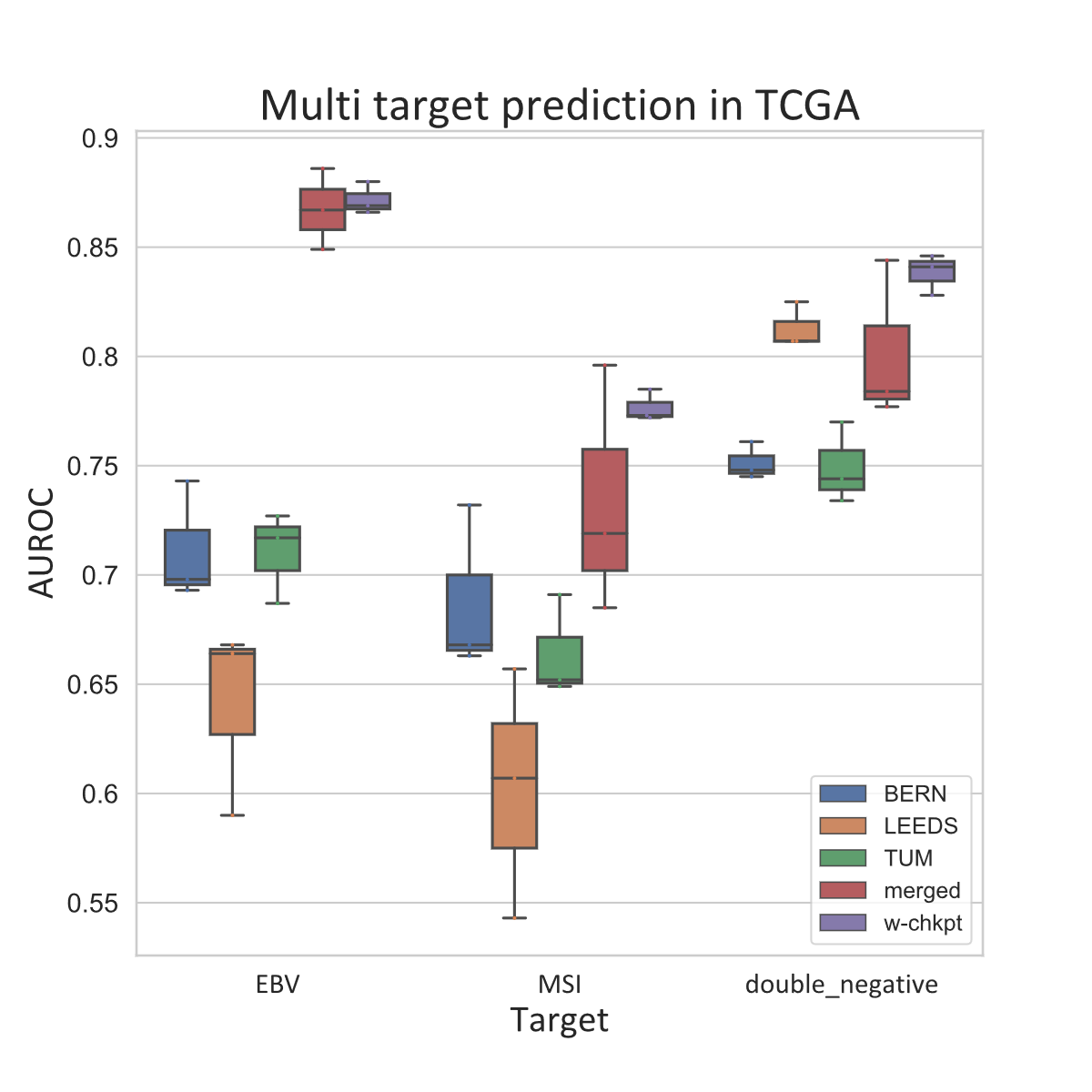
**

**Suppl. Figure 13: Multi target (EBV, MSI and double negative) status prediction from pathology images in gastric cancer with swarm learning.** Classification performance (area under the receiver operating curve, AUROC) for prediction of multi-target status on a patient level in the TCGA cohort. The results of three replicates per experiment are shown as a box plot. The box shows the median and quartiles as the whiskers expand to the rest of the distribution, with the exception of points identified as outliers.

# Supplementary Table Legends

*[Separate file: MSI_EBV_Suppl_Table_1.xlsx]*

Suppl. Table 1: A table containing all raw results of our experimental runs.
